# Supplementary material for: Hand choice is unaffected by high frequency continuous theta burst transcranial magnetic stimulation to the posterior parietal cortex
Source: PLoS One. 2022 Oct 13;17(10):e0275262. doi: 10.1371/journal.pone.0275262 (PMC9560494; doi:10.1371/journal.pone.0275262)
Supplement: S1 File — Hand choice. (DOCX) [file pone.0275262.s001.docx]

**Supplementary materials**

**S1. Supplementary statistical analyses. Hand Choice.**

**Table S1. Hand choice**

(S1.1) Full dataset (N = 26)

***One-way ANOVA:***

*PSE per stimulation condition: F (2, 50) = 1.73, p = 0.19*

*PSE per stimulation condition with No-cTBS: F (3, 75) = 1.58, p = 0.20*

*Proportion RHU per stimulation condition: F (2, 50) = 1.05, p = 0.36*

*Proportion RHU per stimulation condition with No-cTBS: F (3, 75) = 0.98, p = 0.41*

*Proportion RHU at Sham-PSE per stimulation condition: F (2, 50) = 1.23, p = 0.30*

*Proportion RHU at No-cTBS-PSE per stimulation condition with No-cTBS: F (3, 75) = 3.35, p = 0.02*

(S1.2) Left-handers removed (N = 23)

***One-way ANOVA:***

*PSE per stimulation condition: F (2, 44) = 1.44, p = 0.25*

*PSE per stimulation condition with No-cTBS: F (3, 66) = 1.32, p = 0.28*

*Proportion RHU per stimulation condition: F (2, 44) = 0.97, p = 0.39*

*Proportion RHU per stimulation condition with No-cTBS: F (3, 66) = 0.91, p = 0.44*

*Proportion RHU at Sham-PSE per stimulation condition: F (2, 44) = 1.41, p = 0.26*

*Proportion RHU at No-cTBS-PSE per stimulation condition with No-cTBS: F (3, 66) = 3.38, p = 0.02*

(S1.3) Right-handers with strategy removed (N = 24)

***One-way ANOVA:***

*PSE per stimulation condition: F (2, 46) = 1.57, p = 0.22*

*PSE per stimulation condition with No-cTBS: F (3, 69) = 1.40, p = 0.25*

*Proportion RHU per stimulation condition: F (2, 46) = 0.99, p = 0.38*

*Proportion RHU per stimulation condition with No-cTBS: F (3, 69) = 0.94, p = 0.43*

*Proportion RHU at Sham-PSE per stimulation condition: F (2, 46) = 0.99, p = 0.38*

*Proportion RHU at No-cTBS-PSE per stimulation condition with No-cTBS: F (3, 69) = 3.07, p = 0.03*

(S1.4) TMS-averse removed (N = 25)

***One-way ANOVA:***

*PSE per stimulation condition: F (2, 48) = 1.63, p = 0.21*

*PSE per stimulation condition with No-cTBS: F (3, 72) = 1.50, p = 0.22*

*Proportion RHU per stimulation condition: F (2, 48) = 0.99, p = 0.38*

*Proportion RHU per stimulation condition with No-cTBS: F (3, 72) = 0.92, p = 0.43*

*Proportion RHU at Sham-PSE per stimulation condition: F (2, 48) = 1.18, p = 0.32*

*Proportion RHU at No-cTBS-PSE per stimulation condition with No-cTBS: F (3, 72) = 3.20, p = 0.03*

(S1.5) Right-handers, no strategy (N = 20)

***One-way ANOVA:***

*PSE per stimulation condition: F (2, 38) = 1.19, p = 0.31*

*PSE per stimulation condition with No-cTBS: F (3, 57) = 1.07, p = 0.37*

*Proportion RHU per stimulation condition: F (2, 38) = 0.84, p = 0.44*

*Proportion RHU per stimulation condition with No-cTBS: F (3, 57) = 0.80, p = 0.50*

*Proportion RHU at Sham-PSE per stimulation condition: F (2, 38) = 1.09, p = 0.35*

*Proportion RHU at No-cTBS-PSE per stimulation condition with No-cTBS: F (3, 57) = 2.96, p = 0.04*

(S1.6) Right-handers, no strategy, outlier removed (N = 19)

***One-way ANOVA:***

*PSE per stimulation condition: F (2, 36) = 0.56, p = 0.58*

*PSE per stimulation condition with No-cTBS: F (3, 54) = 0.48, p = 0.70*

*Proportion RHU per stimulation condition: F (2, 36) = 0.71, p = 0.50*

*Proportion RHU per stimulation condition with No-cTBS: F (3, 54) = 0.60, p = 0.62*

*Proportion RHU at Sham-PSE per stimulation condition: F (2, 36) = 1.26, p = 0.30*

*Proportion RHU at No-cTBS-PSE per stimulation condition with No-cTBS: F (3, 54) = 2.51, p = 0.07*
