# Supplementary material for: Hand choice is unaffected by high frequency continuous theta burst transcranial magnetic stimulation to the posterior parietal cortex
Source: PLoS One. 2022 Oct 13;17(10):e0275262. doi: 10.1371/journal.pone.0275262 (PMC9560494; doi:10.1371/journal.pone.0275262)
Supplement: S2 File — Response times. (DOCX) [file pone.0275262.s002.docx]

**Supplementary materials**

**S2. Supplementary statistical analyses. Response Times.**

**Table S2. Response times**

(S2.1) Full dataset (N = 26)

***Two-way ANOVA:* Hand x Stimulation condition**

*Main effect of Hand: F (1, 25) = 1.83, p = 0.19*

*Main effect of Stimulation condition: F (2, 50) = 0.19, p = 0.83*

*Interaction term: F (2, 50) = 0.38, p = 0.69*

***Choice costs***

**Space x Stimulation condition: Sham-PSE**

*Main effect of Space: F (1, 25) = 18.33, p = 0.0002*

*Main effect of Stimulation condition: F (2, 50) = 0.11, p = 0.89*

*Interaction term: F (2, 50) = 1.40, p = 0.26*

**Space x Stimulation condition: No-cTBS-PSE**

*Main effect of Space: F (1, 25) = 16.51, p = 0.0004*

*Main effect of Stimulation condition: F (2, 50) = 0.18, p = 0.84*

*Interaction term: F (2, 50) = 1.42, p = 0.25*

(S2.2) Left-handers removed (N = 23)

***Two-way ANOVA:* Hand x Stimulation condition**

*Main effect of Hand: F (1, 22) = 0.82, p = 0.38*

*Main effect of Stimulation condition: F (2, 44) = 0.86, p = 0.43*

*Interaction term: F (2, 44) = 0.10, p = 0.90*

***Choice costs***

**Space x Stimulation condition: Sham-PSE**

*Main effect of Space: F (1, 22) = 15.10, p = 0.0008*

*Main effect of Stimulation condition: F (2, 44) = 0.66, p = 0.52*

*Interaction term: F (2, 44) = 1.82, p = 0.17*

**Space x Stimulation condition: No-cTBS-PSE**

*Main effect of Space: F (1, 22) = 14.95, p = 0.0008*

*Main effect of Stimulation condition: F (2, 44) = 0.33, p = 0.72*

*Interaction term: F (2, 44) = 0.69, p = 0.51*

(S2.3) Right-handers with strategy removed (N = 24)

***Two-way ANOVA:* Hand x Stimulation condition**

*Main effect of Hand: F (1, 23) = 2.50, p = 0.13*

*Main effect of Stimulation condition: F (2, 46) = 0.21, p = 0.81*

*Interaction term: F (2, 46) = 0.18, p = 0.84*

***Choice costs***

**Space x Stimulation condition: Sham-PSE**

*Main effect of Space: F (1, 23) = 15.68, p = 0.0006*

*Main effect of Stimulation condition: F (2, 46) = 0.15, p = 0.86*

*Interaction term: F (2, 46) = 0.87, p = 0.43*

**Space x Stimulation condition: No-cTBS-PSE**

*Main effect of Space: F (1, 23) = 13.96, p = 0.001*

*Main effect of Stimulation condition: F (2, 46) = 0.19, p = 0.83*

*Interaction term: F (2, 46) = 0.90, p = 0.41*

(S2.4) TMS-averse removed (N = 25)

***Two-way ANOVA:* Hand x Stimulation condition**

*Main effect of Hand: F (1, 24) = 1.48, p = 0.24*

*Main effect of Stimulation condition: F (2, 48) = 0.13, p = 0.88*

*Interaction term: F (2, 48) = 0.36, p = 0.70*

***Choice costs***

**Space x Stimulation condition: Sham-PSE**

*Main effect of Space: F (1, 24) = 16.71, p = 0.0004*

*Main effect of Stimulation condition: F (2, 48) = 0.06, p = 0.94*

*Interaction term: F (2, 48) = 1.51, p = 0.23*

**Space x Stimulation condition: No-cTBS-PSE**

*Main effect of Space: F (1, 24) = 14.99, p = 0.0007*

*Main effect of Stimulation condition: F (2, 48) = 0.11, p = 0.90*

*Interaction term: F (2, 48) = 1.62, p = 0.21*

(S2.5) Right-handers, no strategy (N = 20)

***Two-way ANOVA:* Hand x Stimulation condition**

*Main effect of Hand: F (1, 19) = 1.05, p = 0.32*

*Main effect of Stimulation condition: F (2, 38) = 0.85, p = 0.43*

*Interaction term: F (2, 38) = 0.07, p = 0.93*

***Choice costs***

**Space x Stimulation condition: Sham-PSE**

*Main effect of Space: F (1, 19) = 11.02, p = 0.004*

*Main effect of Stimulation condition: F (2, 38) = 0.64, p = 0.53*

*Interaction term: F (2, 50) = 1.24, p = 0.30*

**Space x Stimulation condition: No-cTBS-PSE**

*Main effect of Space: F (1, 19) = 12.90, p = 0.002*

*Main effect of Stimulation condition: F (2, 38) = 0.71, p = 0.50*

*Interaction term: F (2, 50) = 0.82, p = 0.45*

(S2.6) Right-handers, no strategy, outlier removed (N = 19)

***Two-way ANOVA:* Hand x Stimulation condition**

*Main effect of Hand: F (1, 18) = 0.73, p = 0.40*

*Main effect of Stimulation condition: F (2, 36) = 0.90, p = 0.42*

*Interaction term: F (2, 36) = 0.41, p = 0.66*

***Choice costs***

**Space x Stimulation condition: Sham-PSE**

*Main effect of Space: F (1, 18) = 9.05, p = 0.008*

*Main effect of Stimulation condition: F (2, 36) = 0.72, p = 0.49*

*Interaction term: F (2, 36) = 0.67, p = 0.52*

**Space x Stimulation condition: No-cTBS-PSE**

*Main effect of Space: F (1, 18) = 10.75, p = 0.004*

*Main effect of Stimulation condition: F (2, 36) = 0.79, p = 0.46*

*Interaction term: F (2, 36) = 0.26, p = 0.77*
