# Supplementary material for: Hand choice is unaffected by high frequency continuous theta burst transcranial magnetic stimulation to the posterior parietal cortex
Source: PLoS One. 2022 Oct 13;17(10):e0275262. doi: 10.1371/journal.pone.0275262 (PMC9560494; doi:10.1371/journal.pone.0275262)
Supplement: S3 File — (DOCX) [file pone.0275262.s003.docx]

**Supplementary materials**

**S3. Participant errors**

*Table S3. Participant errors.*

| **Participant** | **Total** | **Percent of Participant’s trials** | **Negative response time** | **Double button response** |
| --- | --- | --- | --- | --- |
| **1** | 2 | 0.10 | 0 | 2 |
| **2** | 15 | 0.75 | 3 | 13 |
| **3** | 10 | 0.50 | 9 | 1 |
| **4** | 6 | 0.30 | 2 | 4 |
| **5** | 12 | 0.59 | 3 | 9 |
| **6** | 7 | 0.35 | 4 | 3 |
| **7** | 14 | 0.69 | 4 | 10 |
| **8** | 17 | 0.84 | 13 | 4 |
| **9** | 13 | 0.64 | 6 | 7 |
| **10** | 15 | 0.74 | 4 | 11 |
| **11** | 6 | 0.30 | 0 | 6 |
| **12** | 12 | 0.60 | 7 | 5 |
| **13** | 13 | 0.64 | 7 | 6 |
| **14** | 1 | 0.05 | 1 | 0 |
| **15** | 22 | 1.09 | 12 | 10 |
| **16** | 7 | 0.35 | 2 | 5 |
| **17** | 6 | 0.30 | 3 | 3 |
| **18** | 7 | 0.35 | 4 | 3 |
| **19** | 9 | 0.45 | 7 | 2 |
| **20** | 11 | 0.55 | 7 | 4 |
