# Supplementary material for: Hand choice is unaffected by high frequency continuous theta burst transcranial magnetic stimulation to the posterior parietal cortex
Source: PLoS One. 2022 Oct 13;17(10):e0275262. doi: 10.1371/journal.pone.0275262 (PMC9560494; doi:10.1371/journal.pone.0275262)
Supplement: S5 File — (DOCX) [file pone.0275262.s005.docx]

**Supplementary materials**

**S5. Comparison of reaching studies**

A synthesis of reported prior neuroimaging and TMS results involving reaching and pointing is presented in Figure 5 of the paper. Specifically, we used the following method to provide an objective estimate of the overlap in reported effects across studies. We take the reported coordinates of peak activation (fMRI/PET) or target area (TMS coil localisation) and create a 15mm-diameter spherical foci of ‘activity’ around these coordinates, as the centroid. We then use these projected ‘activations’ to compute a heat map representing the percent overlap between studies, projected on the three-dimensional cortical surface of a single individual in standardised (Talairach) space. Foci with a minimum of 20% overlap with other coordinates are illustrated. Our choice of 15mm-diameter foci with ≥20% overlap to represent the variability, or noise, in the location of these reported coordinates was otherwise arbitrary. A total of 14 fMRI, three PET, and 7 TMS studies were included. Where necessary, reported MNI coordinates were transformed to Talairach coordinates [1], using the approach provided by the Cambridge Brain Sciences Unit [2]. This provides a simple way to visualise the variation/consistency in reported functional-localisation data from prior studies involving reaching and arm movements alongside the coordinates we used as a guide for TMS coil localisation in the current study, based on our own recent fMRI results [3].

**References**

1. Talairach J, Tournoux P. Co-planar stereotaxic atlas of the human brain-3-dimensional proportional system. New York: Thieme Medical Publishers; 1988.

2. Brett M. The MNI brain and the Talairach atlas [Internet]. 2017. Available from: http://imaging.mrc-cbu.cam.ac.uk/imaging/MniTalairach

3. Fitzpatrick AM, Dundon NM, Valyear KF. The neural basis of hand choice: An fMRI investigation of the Posterior Parietal Interhemispheric Competition model. Neuroimage. 2019 Jan 15;185:208–21.

**Comparison of reaching studies:**

Astafiev, S. V, Shulman, G. L., Stanley, C. M., Snyder, A. Z., Van Essen, D. C., & Corbetta, M. (2003). Functional organization of human intraparietal and frontal cortex for attending, looking, and pointing. *Journal of Neuroscience*, *23*(11), 4689–4699.

Blangero, A., Menz, M. M., McNamara, A., & Binkofski, F. (2009). Parietal modules for reaching. *Neuropsychologia, 47*(6), 1500–1507.

Cavina-Pratesi, C., Monaco, S., Fattori, P., Galletti, C., McAdam, T. D., Quinlan, D. J., Goodale, M. A., & Culham, J. C. (2010). Functional magnetic resonance imaging reveals the neural substrates of arm transport and grip formation in reach-to-grasp actions in humans. *Journal of Neuroscience*, *30*(31), 10306–10323.

Connolly, J. D., Goodale, M. A., Desouza, J. F., Menon, R. S., Vilis, T., & Medical Research Council Group for Action and Perception). (2000). A comparison of frontoparietal fMRI activation during anti-saccades and anti-pointing. *Journal of Neurophysiology, 84(*3), 1645-1655.

Connolly, J. D., Andersen, R. A., & Goodale, M. A. (2003). FMRI evidence for a “parietal reach region” in the human brain. *Experimental Brain Research*, *153*(2), 140–145.

Davare, M., Zénon, A., Desmurget, M., & Olivier, E. (2015). Dissociable contribution of the parietal and frontal cortex to coding movement direction and amplitude. *Frontiers in Human Neuroscience, 9*, 241.

De Jong, B. M., Van der Graaf, F. H. C. E., & Paans, A. M. J. (2001). Brain activation related to the representations of external space and body scheme in visuomotor control. *NeuroImage, 14*(5), 1128–1135.

Desmurget, M., Epstein, C. M., Turner, R. S., Prablanc, C., Alexander, G. E., & Grafton, S. T. (1999). Role of the posterior parietal cortex in updating reaching movements to a visual target. *Nature Neuroscience*, *2*(6), 563.

Desmurget, M., Gréa, H., Grethe, J. S., Prablanc, C., Alexander, G. E., & Grafton, S. T. (2001). Functional anatomy of nonvisual feedback loops during reaching: a positron emission tomography study. *Journal of Neuroscience, 21*(8), 2919–2928.

Fernandez-Ruiz, J., Goltz, H. C., DeSouza, J. F. X., Vilis, T., & Crawford, J. D. (2007). Human parietal "reach region" primarily encodes intrinsic visual direction, not extrinsic movement direction, in a visual--motor dissociation task. *Cerebral Cortex*, *17*(10), 2283–2292.

Filimon, F., Nelson, J. D., Hagler, D. J., & Sereno, M. I. (2007). Human cortical representations for reaching: mirror neurons for execution, observation, and imagery. *NeuroImage, 37*(4), 1315–1328.

Gallivan, J. P., McLean, D. A., Smith, F. W., & Culham, J. C. (2011). Decoding effector-dependent and effector-independent movement intentions from human parieto-frontal brain activity. *Journal of Neuroscience*, *31*(47), 17149–17168.

Glover, S., Miall, R. C., & Rushworth, M. F. S. (2005). Parietal rTMS disrupts the initiation but not the execution of on-line adjustments to a perturbation of object size. *Journal of Cognitive Neuroscience*, *17*(1), 124–136.

Hinkley, L. B. N., Krubitzer, L. A., Padberg, J., & Disbrow, E. A. (2009). Visual-manual exploration and posterior parietal cortex in humans. *Journal of Neurophysiology*, *102*(6), 3433–3446.

Inoue, K., Kawashima, R., Satoh, K., Kinomura, S., Goto, R., Koyama, M., Sugiura, M., Ito, M., & Fukuda, H. (1998). PET study of pointing with visual feedback of moving hands. *Journal of Neurophysiology, 79*(1), 117–125.

Kertzman, C., Schwarz, U., Zeffiro, T. A., & Hallett, M. (1997). The role of posterior parietal cortex in visually guided reaching movements in humans. *Experimental Brain Research, 114*(1), 170–183.

Konen, C. S., Mruczek, R. E., Montoya, J. L., & Kastner, S. (2013). Functional organization of human posterior parietal cortex: grasping-and reaching-related activations relative to topographically organized cortex. J*ournal of Neurophysiology, 109*(12), 2897–2908.

Le, A., Vesia, M., Yan, X., Crawford, J. D., & Niemeier, M. (2016). Parietal area BA7 integrates motor programs for reaching, grasping, and bimanual coordination. *Journal of Neurophysiology, 117*(2), 624–636.

Medendorp, W. P., Goltz, H. C., Vilis, T., & Crawford, J. D. (2003). Gaze-centered updating of visual space in human parietal cortex. *Journal of Neuroscience*, *23*(15), 6209–6214.

Pellijeff, A., Bonilha, L., Morgan, P. S., McKenzie, K., & Jackson, S. R. (2006). Parietal updating of limb posture: an event-related fMRI study. *Neuropsychologia, 44*(13), 2685–2690.

Prado, J., Clavagnier, S., Otzenberger, H., Scheiber, C., Kennedy, H., & Perenin, M.-T. (2005). Two cortical systems for reaching in central and peripheral vision. *Neuron*, *48*(5), 849–858.

Reichenbach, A., Bresciani, J. P., Peer, A., Bülthoff, H. H., & Thielscher, A. (2010). Contributions of the PPC to online control of visually guided reaching movements assessed with fMRI-guided TMS. *Cerebral Cortex, 21*(7), 1602–1612.

Striemer, C. L., Chouinard, P. A., & Goodale, M. A. (2011). Programs for action in superior parietal cortex: a triple-pulse TMS investigation. *Neuropsychologia*, *49*(9), 2391–2399.

Vesia, M., Prime, S. L., Yan, X., Sergio, L. E., & Crawford, J. D. (2010). Specificity of human parietal saccade and reach regions during transcranial magnetic stimulation. *Journal of Neuroscience*, *30*(39), 13053–13065.
