# Supplementary material for: Hand choice is unaffected by high frequency continuous theta burst transcranial magnetic stimulation to the posterior parietal cortex
Source: PLoS One. 2022 Oct 13;17(10):e0275262. doi: 10.1371/journal.pone.0275262 (PMC9560494; doi:10.1371/journal.pone.0275262)
Supplement: S6 File — (DOCX) [file pone.0275262.s006.docx]

**Supplementary materials**

**S6. Post-stimulation questionnaire data**

*Table S6.1.* Post-stimulation questionnaire data. Full dataset (N = 26)

| **A.** | | | | |
| --- | --- | --- | --- | --- |
| **Type of Session** | **Reported as Real** | **Reported as Sham** | **Reported “I don’t know”** | **Percent correct identification** |
|  |  |  |  | *Total sample \| Respondents* |
| **Real cTBS** | 28 | 5 | 19 | 53.85% \| 84.85% |
| **Sham cTBS** | 13 | 2 | 11 | 7.69% \| 13.33% |
|  |  |  |  |  |
| **B.** | | | | |
| **Type of Sensation** | **Reported after Real** | | **Reported after Sham** | |
|  |  |  |  |  |
| **Itching** | 6 | | 1 | |
| **Pain** | 6 | | 1 | |
| **Burning** | - | | 1 | |
| **Warmth/heat** | 7 | | 4 | |
| **Pinching** | 6 | | 1 | |
| **Metallic/iron taste** | 2 | | - | |
| **Fatigue** | 8 | | 4 | |
| **Dazed** | 1 | | - | |
| **Tapping** | 4 | | 1 | |
| **Twitching** | 1 | | - | |
|  |  |  |  | |

*Table S6.2.* Post-stimulation questionnaire data. TMS-averse removed (N = 25)

| **A.** | | | | |
| --- | --- | --- | --- | --- |
| **Type of Session** | **Reported as Real** | **Reported as Sham** | **Reported “I don’t know”** | **Percent correct identification** |
|  |  |  |  | *Total sample \| Respondents* |
| **Real cTBS** | 26 | 5 | 19 | 52.00% \| 81.25% |
| **Sham cTBS** | 12 | 2 | 11 | 8.00% \| 14.29% |
|  |  |  |  |  |
| **B.** | | | | |
| **Type of Sensation** | **Reported after Real** | | **Reported after Sham** | |
|  |  |  |  |  |
| **Itching** | 5 | | 1 | |
| **Pain** | 4 | | - | |
| **Burning** | - | | - | |
| **Warmth/heat** | 5 | | 3 | |
| **Pinching** | 5 | | - | |
| **Metallic/iron taste** | - | | - | |
| **Fatigue** | 6 | | 3 | |
| **Dazed** | 1 | | - | |
| **Tapping** | 4 | | 1 | |
| **Twitching** | 1 | | - | |
|  |  |  |  | |

*Table S6.3.* Post-stimulation questionnaire data. Right-handers, no strategy (N = 20)

| **A.** | | | | |
| --- | --- | --- | --- | --- |
| **Type of Session** | **Reported as Real** | **Reported as Sham** | **Reported “I don’t know”** | **Percent correct identification** |
|  |  |  |  | *Total sample \| Respondents* |
| **Real cTBS** | 19 | 4 | 17 | 47.50% \| 82.61% |
| **Sham cTBS** | 10 | 0 | 10 | 0% |
|  |  |  |  |  |
| **B.** | | | | |
| **Type of Sensation** | **Reported after Real** | | **Reported after Sham** | |
|  |  |  |  |  |
| **Itching** | 5 | | 1 | |
| **Pain** | 1 | | - | |
| **Burning** | - | | - | |
| **Warmth/heat** | 3 | | 2 | |
| **Pinching** | 4 | | - | |
| **Metallic/iron taste** | - | | - | |
| **Fatigue** | 4 | | 1 | |
| **Dazed** | 1 | | - | |
| **Tapping** | 4 | | 1 | |
| **Twitching** | 5 | | 1 | |
|  |  |  |  | |
