# Supplementary material for: Hand choice is unaffected by high frequency continuous theta burst transcranial magnetic stimulation to the posterior parietal cortex
Source: PLoS One. 2022 Oct 13;17(10):e0275262. doi: 10.1371/journal.pone.0275262 (PMC9560494; doi:10.1371/journal.pone.0275262)
Supplement: Attachment — Submitted filename: PONE-D-22-13883 - Review.pdf [file pone.0275262.s007.pdf]

PONE-D-22-13883

Review

General comments

The manuscript is very well written, with commendable fluency, straightness and assertiveness. Although the results were not those predicted by the authors, they were clear in their description, providing the tools for the reader to understand the study and the possible reasons for the results obtained. The study, therefore, is of great relevance for understanding the differences between current forms of non-invasive brain stimulation, namely, transcranial magnetic stimulation (TMS), high-frequency repetitive continuous theta burst stimulation (cTBS) and transcranial direct current stimulation (tDCS). It also contributes to the discussion about the parameters of measurement and application of cTBS, particularly when applied to regions not directly related to the primary motor cortex.

I only have few suggestions for the manuscript. I present them below, separated by section of the manuscript.

Introduction

I enjoyed reading the introduction, although I missed a greater number of references - or a brief discussion about the scarcity of productions related to the topic. The development of the introduction is well done, fluid and dynamic. The presentation of the model idealized by the authors is well-organized and sufficiently detailed.

In lines 58-62, you cite two models based on competition between two neuronal populations. The excerpt that extends from line 62 to line 69, however, makes several important claims, but there are no references to them. If this excerpt is an explanation of the models cited in lines 58-62, it is necessary to mention the link between the two excerpts.

Methods

The inclusion of the work hypotheses on the aspredicted.org portal is an interesting work strategy that should be endorsed, as well the sample sizing through a free and accessible tool.

The use of the Waterloo Handedness Inventory is interesting. According to the article cited, "*the type of questionnaire used in the present investigation allows subjects to indicate both the amount or degree of their hand preference and the direction of their hand preference*" (1). Did you rate the degree of hand preference (consistency) of the participants? Although Steenhuis et colleagues (1) comments that the population's manuality consistency is usually high, I would like to know if your sample was homogeneous in terms of consistency and if the left-handers in the study had a high or low manuality consistency.

The description of the stimulation protocol is well done and detailed. As the two articles used to justify the positioning of the coil-handle are from the same research group and quite old – 2008 and 2010 – I believe that only the second reference is enough, or I suggest that the second is kept and a more recent reference is added.

Regarding the behavioral test, Oliveira et al. (2) state that "*the instructions emphasized that the responses should be initiated and completed as fast as possible in a single smooth movement, and that end-point errors need not be corrected*". Was there a similar instruction in your study?

Completing my 'Methods' commentaries, there is a significant change in the design of the work compared to the work developed by Oliveira (2) and Valyear (3) and collaborators, which now uses a touchscreen monitor and different angles of stimulus presentation, although the difference is minimal. I believe you could briefly explain why you chose to change the angles in relation to the studies cited.

Results

The results are well described and organized, and their presentation is clear and easily understandable. I only have two comments about the Figures 4 and 5.

Figure 4 does not clearly illustrate the difference between the RTs of targets close to the PSE and in extreme positions, although it provides more information about the data distribution. I think the representation of the difference between the RTs in the two conditions benefits more from another graphical representation strategy. The inset does not adequately illustrate the collapsed difference between the RTs, and does not provide units that allow its dimensioning..

Figure 5 is very good, but the green line that connects the highlighted region to the highlight frame in the posterior and supero-posterior views interferes with the visualization. I suggest also delimiting the highlighted region and connect it to the highlight frame with solid lines at the vertices, demonstrating the applied zoom.

Discussion

The discussion follows the same pattern of organization and quality of the manuscript. The authors are thorough in analyzing the possible causes of the differences found between their study and similar studies that preceded it. They assess the impact that the form of stimulation (cTBS, tDCS, sp-TMS) may have caused, probably constituting the main responsible for the difference between the results obtained, while considering the possible effects caused by the sample and the study design.

The influence of the vision in the previous studies and in the current study also seems to be relevant and, if it was not an important factor allied to the the difference in results, it was a factor of divergence between them, which makes the similarities and differences between their results less comparable. Thus, it would be interesting to contemplate a little more in the discussion the bias that the vision may have brought to the study results.

References

1. Steenhuis, R. E., & Bryden, M. P. (1989). Different dimensions of hand preference that relate to skilled and unskilled activities. Cortex; a journal devoted to the study of the nervous system and behavior, 25(2), 289–304. https://doi.org/10.1016/s0010-9452(89)80044-9
2. Oliveira, F. T., Diedrichsen, J., Verstynen, T., Duque, J., & Ivry, R. B. (2010). Transcranial magnetic stimulation of posterior parietal cortex affects decisions of hand choice. Proceedings of the

National Academy of Sciences of the United States of America, 107(41), 17751–17756. https://doi.org/10.1073/pnas.1006223107

3. Valyear, K. F., Fitzpatrick, A. M., & Dundon, N. M. (2019). Now and then: Hand choice is influenced by recent action history. Psychonomic bulletin & review, 26(1), 305–314. https://doi.org/10.3758/s13423-018-1510-1
